# Hernia of Umbilical Cord: Report of Three Unusual Cases

**Published:** 2015-04-01

**Authors:** Bilal Mirza, Afzal Mirza, Imran Hashim, Muhammad Saleem

**Affiliations:** Department of Pediatric Surgery, The Children’s Hospital and the Institute of Child Health, Lahore, Pakistan

**Keywords:** Hernia of umbilical cord, Evisceration of bowel, Patent vitellointestinal duct, Neonatal intestinal obstruction, Intestinal atresia, Colonic stenosis

## Abstract

Congenital hernia of umbilical cord is a less frequent entity in newborns and occasionally associated with other maladies. Herein, we report three unusual cases of hernia of umbilical cord. First case was associated with in-utero evisceration of entire small bowel through the presumably ruptured hernia of umbilical cord and other two cases had associated patent vitellointestinal duct (PVID). All of the cases were managed successfully.

## CASE REPORT

**Case 1:**

 A term female newborn, weighing 2.5 kg and product of non-consanguineous marriage, presented with bowel evisceration through the umbilical cord. The patient was delivered at home by spontaneous vaginal delivery. No antenatal scans were performed. The patient was given initial resuscitation in terms of covering the bowel with warm saline soaked gauzes, maintaining an IV line and infusing IV fluids, prophylactic antibiotics were started, and baseline work-up was sent. 

The patient was thought of gastroschisis on initial presentation; however, thorough examination in the operation theatre divulged bowel evisceration through the umbilical cord which was attached normally to the umbilical ring; there was no abdominal wall defect. A 1.5 cm strip of skin was covering the proximal aspect of the umbilical cord and ring (Fig. 1). The eviscerated bowel was viable, thick walled, and covered with a fibrinous peel as seen in cases of gastroschisis. The umbilicus was explored and found a patent umbilical ring which was incised right transversally to extend the wound for further exploration. The peel was dissected meticulously and found entire small bowel up to transverse colon in it. There was an exit level (type I) atresia at proximal jejunum (Fig. 2); at entry level of the umbilical cord defect, there was a 3cm long stenosis of the ascending colon with a mesenteric defect (Fig. 3). Associated malrotation of the gut was also present. Resection of dilated jejunum and Jejuno-jejunal anastomosis was performed just few cm distal to the DJ junction and a colostomy is formed at the level of ascending colon stenosis. 

**Figure:**
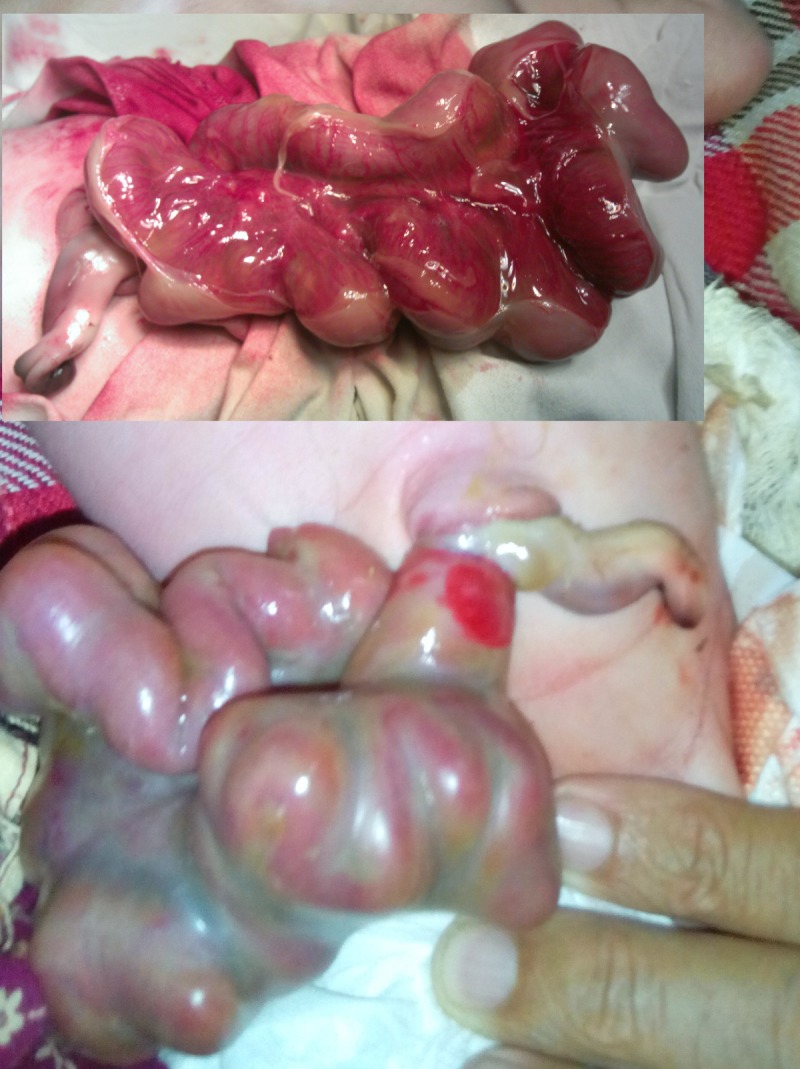
Figure 1: Bowel evisceration through the umbilical cord. Inset shows meticulous dissection of the matted loops.

**Figure:**
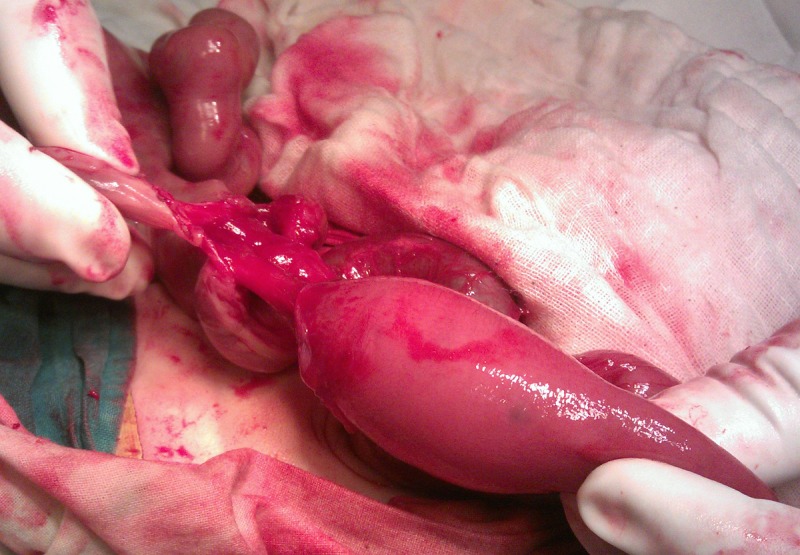
Figure 2: Type I jejunal atresia at exit level.

**Figure:**
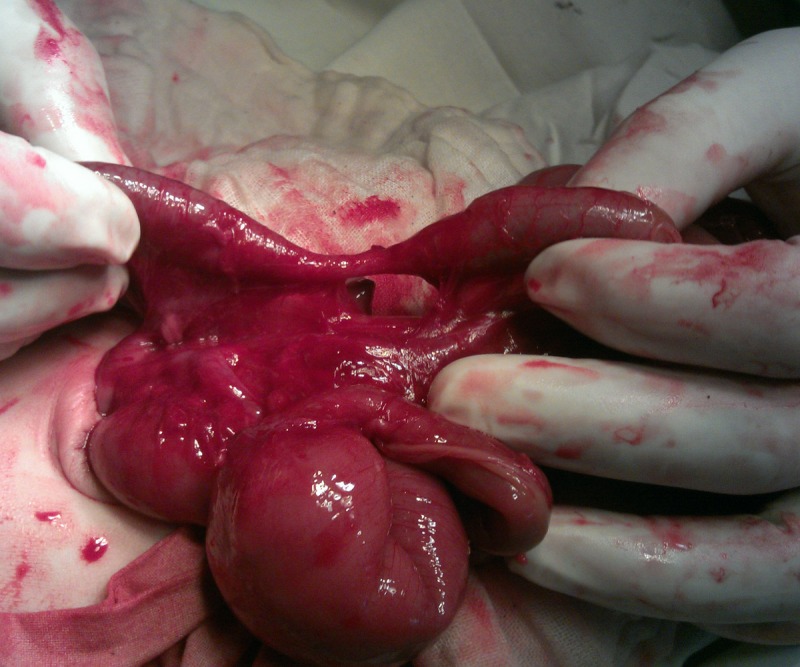
Figure 3: Long ascending colon stenosis with a mesenteric defect. Proximal jejunal atresia can also be seen.

**Case 2:**

A male term newborn, weighing 2.7kg product of consanguineous marriage and delivered by spontaneous vaginal delivery, presented with a mass at umbilicus. After newborn resuscitation, the entity was carefully examined and found to have a normally inserted umbilicus with a loop of bowel visible at the base of umbilical cord and some bowel mucosa appeared extruded from a side of umbilical cord. The proximal portion of cord and umbilical ring were covered with 1cm strip of normal skin (Fig. 4). The extruded mucosa had two openings and meconium was being passed from one opening. The patient was vitally stable and operated electively on next day of admission. At surgery, the umbilical ring was patent with a loop of small bowel herniated through the ring to the base of umbilical cord. The extruded mucosa was mobilized off the umbilical cord and found to be a prolapsing PVID which was resected and an end-to-end ileo-ileal anastomosis performed. The postoperative recovery was uneventful. The patient was discharged on 5th postoperative day in a good clinical condition.

**Figure:**
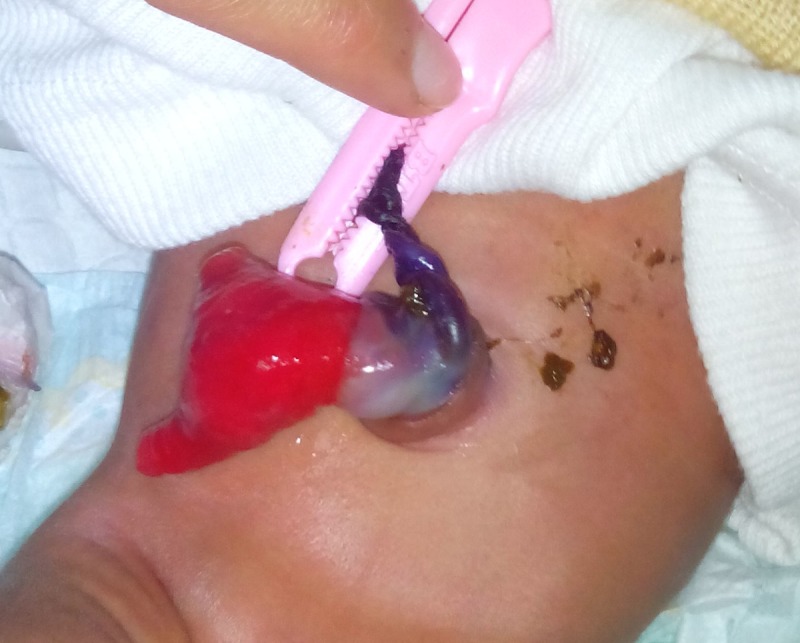
Figure 4: Hernia of umbilical cord with prolapsing PVID.

**Case 3:**

A male term newborn, weighing 2.9kg, presented with a mass at mid abdomen. The perinatal events were normal. On examination the umbilicus was normally sited without any abdominal wall defect. The umbilical ring was patent and bowel loops were herniated into the umbilical cord and covered with a sac as found in omphalocele. A strip of 2.5cm was encircling the base of umbilicus. The newborn passed stool from a mucosal tube extruded from the umbilical mass (Fig. 5). Initial diagnosis was omphalocele however detailed inspection made it clear as hernia of umbilical cord with PVID. At operation, PVID was confirmed and resection and end-to-end ileo-ileal anastomosis done. The contents were reduced to the abdomen and umbilical ring was closed. Postoperative recovery was uneventful. 

**Figure:**
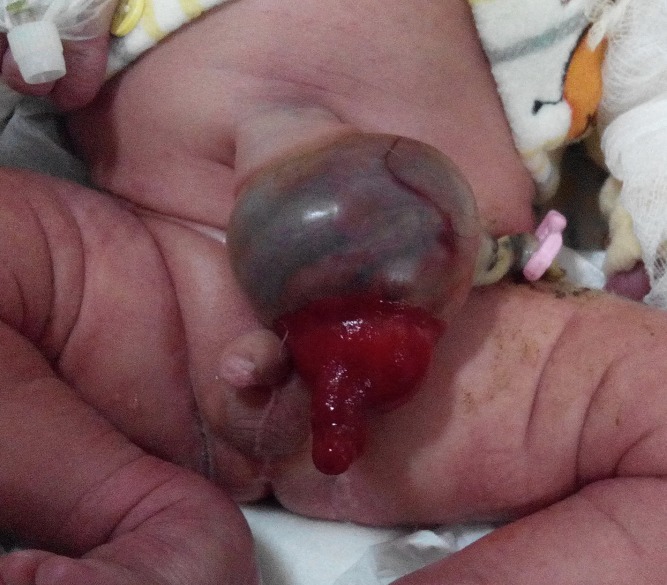
Figure 5: Hernia of umbilical cord with prolapsing PVID.

## DISCUSSION

Congenital hernia of umbilical cord is herniation of small bowel and occasionally other viscera into the umbilical cord on account of failed closure of umbilical ring or failure of complete return of physiologically herniated bowel to the abdomen as opposed to omphalocele minor which is its misnomer and refers to a true abdominal wall deficiency.[1-3] A strip of normal skin always enwraps the umbilical ring and proximal part of the cord which is normally sited in case of hernia of umbilical cord as also featured in all of our cases. Herniation into the umbilical cord may range from a small portion to the entire small bowel along with part of colon.[2,3] in our second case a small part of small bowel was herniated but in third case significant small bowel was herniated into the umbilical cord and simulating it as omphalocele however presence of patent umbilical ring, absence of true abdominal wall defect, and skin coverage of proximal cord and ring helped differentiate it to hernia of umbilical cord. PVID and its remnants may also be found inside the umbilical cord.[4] In our second and third cases, a prolapsing PVID was present. Rarely other abdominal viscera may also herniate into the cord. Hasaniya et al reported a case of incarceration of herniated liver and gallbladder into the umbilical cord.[5] 

During 10-12th week of gestation, the physiologically herniated bowel into the umbilical coelom returns back to the abdomen. During its stay into the umbilical coelom, various issues may occur. There may be a partial failure of return of bowel back to the abdomen as happens in majority of hernia of umbilical cord or it may be an exquisite failure as reported by Gajdhar et al [2]; in their case entire small bowel including cecum and ascending colon were herniated into the cord and covered by a membrane.[2] This is occasionally associated with intestinal atresia.[3] Rarer still, the herniated bowel may distend into the umbilical coelom resulting in its rupture which may prove sinister. Haas et al [1] reported a fetal demise in their series owing to rupture of umbilical cord that led to in-utero bowel evisceration and rupture of umbilical vessels. 

The eviscerated bowel floats freely into the amniotic fluid which may occasionally twist resulting in in-utero volvulus causing loss of bowel and intestinal atresia in the wake. We previously reported a case of congenital short gut and intestinal atresia associated with hernia of umbilical cord; similar in-utero event would have led to this dismal outcome.[6]

The association of intestinal atresia in hernia of umbilical cord may be attributed to hampered blood supply to the bowel. In case of ruptured hernia of umbilical cord, both vascular factors as well as a constricting effect at entry/exit level of cord might be the accusatory factors.[1] This is similar to the closing gastroschisis which is associated with entry/exit level atresia.[7] In our first case, there was type I proximal jejunal atresia at exit level and a long stenosis of the ascending colon at entry level of the defect in the wall of the umbilical cord. The mesenteric defect at the level of ascending colon stenosis and small constricting defect of umbilical cord may be thought as etiology of vascular insufficiency leading to intestinal atresia/stenosis in our first case. 

To infer, though hernia of umbilical cord is a simple manageable anomaly with good outcome; it rarely takes a different path leading to various complications. Mucosal prolapse in PVID is a well-known fact as encountered in our two cases. The association of hernia of umbilical cord with in-utero intestinal evisceration, intestinal atresia, and colonic stenosis is a new addition to the existing literature as on perusal of literature, to best of our knowledge, no similar case could be fetched.

## Footnotes

**Source of Support:** Nil

**Conflict of Interest:** The corresponding author is editor of the journal. The manuscript was independently handled by Editor in chief and author is not involved in decision making of this manuscript.

